# The emergence of metronidazole-resistant *Prevotella bivia* harboring *nimK* gene in Japan

**DOI:** 10.1128/spectrum.00562-24

**Published:** 2024-08-20

**Authors:** Yukitaka Ito, Yusuke Hashimoto, Masato Suzuki, Naomi Kaneko, Mieko Yoshida, Haruo Nakayama, Haruyoshi Tomita

**Affiliations:** 1 Division of Clinical Microbiology Laboratory, Toho University Ohashi Medical Center, Meguro, Tokyo, Japan; 2 Department of Infection and Prevention, Toho University Ohashi Medical Center, Meguro, Tokyo, Japan; 3 Department of Bacteriology, Gunma University Graduate School of Medicine, Maebashi, Gunma, Japan; 4 Antimicrobial Resistance Research Center, National Institute of Infectious Diseases, Higashimurayama, Tokyo, Japan; 5 Laboratory of Bacterial Drug Resistance, Gunma University Graduate School of Medicine, Maebashi, Gunma, Japan; University of Pittsburgh School of Medicine, Pittsburgh, Pennsylvania, USA

**Keywords:** metronidazole, *nimK*, *Prevotella bivia*, whole-genome sequencing (WGS), mobile genetic element (MGE)

## Abstract

We present the identification and characterization of the complete genome of metronidazole (MTZ)-resistant *Prevotella bivia* strain TOH-2715 [minimum inhibitory concentration (MIC): 8 mg/L], isolated from the urine of an elderly Japanese woman, as well as details of its mobile genetic elements (MGEs) containing antimicrobial resistance (AMR) genes and its relationship with other bacterial species determined using whole-genome sequencing (WGS) data. TOH-2715 possessed two chromosomes with putative MGEs containing AMR genes. Two AMR-related MGE regions were present in chromosome 2. MGE-region 1 (7,821 bp) included Tn*6456*, where *nimK* was located, and MGE-region 2 (58.8 Kbp) included the integrative and conjugative element (ICE), where *tet*(Q) and *ermF* were located. The genetic structure of the ICE of TOH-2715 was similar to that of CTn*DOT*-family transposons, where *ermF* and *tet*(Q) are located. A search of public databases revealed that *nimK* was present in *Prevotella* spp., including *P. bivia*, and was partially composed of a Tn*6456*-like element lacking the efflux transporter gene *qacE* and the Crp/Fnr family transcriptional regulator gene in some cases. Core ICE gene analysis showed that ICEs similar to that of TOH-2715 were present in *Prevotella* spp. and *Bacteroides* spp., suggesting horizontal gene transfer among anaerobes. This is the report of WGS analysis of an MTZ-resistant clinical strain of *P. bivia* (TOH-2715) with Tn*6456* encoding *nimK*. Other submitted genomes have described the presence of *nimK*, but none of them have described MTZ resistance. Additionally, we described putative MGE regions containing the AMR gene within the genus *Prevotella* and among anaerobes, raising concerns about the future spread of *nimK* among anaerobes.

**IMPORTANCE:**

Metronidazole (MTZ) is an important antimicrobial agent in anaerobic infections and is widely used in clinical settings. The rate of MTZ resistance in anaerobic bacteria has been increasing in recent years, and the *nim* gene (nitro-imidazole reductase) is one of the resistance mechanisms. *Prevotella bivia* is found in humans in the urinary tract and vagina and is known to cause infections in some cases. One of the *nim* genes, *nimK*, has recently been discovered in this species of bacteria, but there are no reports of antimicrobial resistance (AMR)-related regions in its whole genome level. In this study, we analyzed the AMR region of *nimK*-positive *P. bivia* derived from clinical specimens based on comparisons with other anaerobic genomes. *P. bivia* was found to be engaged in horizontal gene transfer with other anaerobic bacteria, and the future spread of the *nimK* gene is a concern.

## OBSERVATION

*Prevotella* spp. are anaerobic Gram-negative bacilli frequently detected in the oral cavity, respiratory tract, gut, and urogenital tract. Among them, *Prevotella bivia*, originally included in the genus *Bacteroides*, is the most frequently detected in the human urogenital tract (1). *Prevotella* spp. has been reported to occasionally cause urogenital and bloodstream infections. Metronidazole (MTZ) is an effective treatment for *Prevotella* spp. It has been used for over 55 years and is an important antibiotic for managing anaerobic infections, demonstrating rapid bactericidal action against anaerobes (2). Although MTZ resistance in anaerobes has been reported as remaining low, there have been several reports of infections caused by MTZ-resistant anaerobes (3, 4).

The rise in anaerobic bacteria carrying *nim*, which encodes 5-nitroimidazole reductase conferring MTZ resistance, is a significant public health concern. *nim* was initially identified in 1989 as a plasmid-mediated resistance gene conferring MTZ resistance (5, 6). To date, twelve *nim* variants (*nimA* to *nimL*) have been reported, all of which are considered to possess the same function (7, 8). *nim* has been reported in the *Bacteroides* spp., *Fusobacterium* spp., *Prevotella* spp., and *Veillonella* spp. (7). However, the incidence of *Prevotella* spp. carrying *nim* is rare, with only one report of *nimK* on the basis of whole-genome sequencing (WGS) analysis and no other reports based on antimicrobial resistance (AMR) analyses (9). In this study, we elucidated the complete genome of the MTZ-resistant *P. bivia* strain TOH-2715, carrying *nimK*, isolated from urine samples from a hospitalized patient. We also performed a comparative analysis of AMR-related mobile genetic element (MGE) regions.

In December 2022, a woman in her 70s presented to Toho University Ohashi Medical Center with asymptomatic gross hematuria. Urothelial carcinoma (urine cytology class III) and urinary tract infection were suspected. She was treated with levofloxacin (500 mg daily). An anaerobic Gram-negative bacillus (TOH-2715) was isolated from a urine sample collected prior to levofloxacin administration.

Strain TOH-2715 was identified as *P. bivia* using the Vitek2 Compact System (bioMérieux Inc., Hazelwood, MO, USA), with a score of 99%. The minimum inhibitory concentration (MIC) of antimicrobial agents against TOH-2715 was determined using Dry Plate “Eiken” DP53 (Eiken Chemical Co., Ltd., Tochigi, Japan). DP53 is a 96-well plate coated with antimicrobial agents that assesses the antimicrobial susceptibility of anaerobic bacteria using the broth microdilution method. Using the European Committee on Antimicrobial Susceptibility Testing breakpoints, TOH-2715 was found susceptible to all tested β-lactams, including benzylpenicillin (≤0.12 mg/L), ampicillin (≤0.12 mg/L), ampicillin/sulbactam (≤1 mg/L), piperacillin/tazobactam (≤2 mg/L), and meropenem (≤0.25 mg/L). However, it was resistant to MTZ (≥16 mg/L), clindamycin (CLI; ≥4 mg/L), and moxifloxacin (≥4 mg/L) (10). The MIC of MTZ was verified by Etest (bioMérieux Inc.), still as 8 mg/L.

TOH-2715 underwent PCR using universal primers NIM3 (5′-ATGTTCAGAGAAATGCGGCGTAAGCG-3′) and NIM5 (5′-GCTTCCTTGCCTGTCATGTGCTC-3′) to confirm the presence of *nim* genes (458 bp) (11). The PCR amplification products were purified and sequenced, revealing partial *nim* sequences. We determined that the *nim* sequence was most closely related to *nimK* (GenBank accession no. MG827401.1), with a sequence identity of 99.3%.

**Fig 1:**
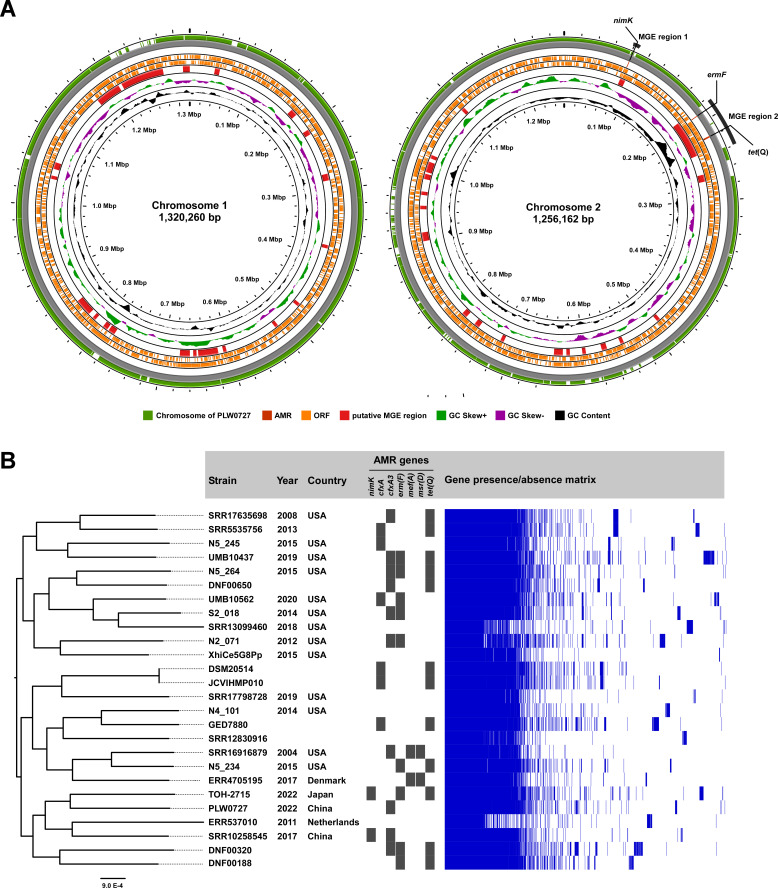
Comparison of the complete genome of *P. bivia* TOH-2715 and core genome analysis using *P. bivia* uncovered from public databases and possession of antimicrobial resistance genes. (**A**) Two chromosomes (chromosome 1 and chromosome 2) of *P. bivia* TOH-2715 are shown. For the inner to outer tracks, GC content (black), GC Skew (purple and green), putative MGE region (red), ORF (orange), AMR gene (dark red), and mapping results for strain PLW0727 (CP126676.1 and CP126677.1) are shown. Putative MGE regions and AMR genes were searched by Alien Hunter and CARD, respectively, and figures were generated by Proksee. (**B**) Core genomes from 26 *P. bivia* genomes found in public databases were identified in Roary and phylogenetic analysis was performed in RAxML. Black panels indicate the possession status of antimicrobial resistance genes identified by CARD, and blue panels indicate gene presence/absence matrix.

We performed WGS to obtain the complete genome of TOH-2715 by hybrid assembly (Fig. 1; Supplemental material). The GC content of TOH-2715 was 39.8%, and it contained two chromosomes (chromosome 1: 1,320,260bp; chromosome 2: 1,256,162bp) and 2,161 predicted coding sequences (CDSs) (Fig. 1A). Average nucleotide identity (ANI) analysis revealed that TOH-2715 had 98.4% sequence identity with *P. bivia* strain JCVIHMP010 (accession no. NZ_ADFO01000000), confirming the species as *P. bivia*. A comparative analysis with the complete genome of *P. bivia* PLW0727, which was first reported in 2023 (12), revealed ANI values of 98.7% and 98.6% for chromosome 1 and chromosome 2, respectively, indicating a high sequence identity. Compared with chromosome 2 of PLW0727, chromosome 2 of TOH-2715 had several unique MGE regions (Fig. 1A). Detection of AMR genes using the Comprehensive Antibiotic Resistance Database revealed *nimK*, *ermF,* and *tet*(Q) in two of these MGE regions, designated as MGE-region 1 and MGE-region 2 (Fig. 1A and B). No AMR genes were detected in chromosome 1 of TOH-2715.

*nimK*, which was located on Tn*6456*, was first reported in the Netherlands in 2018 in a clinical isolate of *P. bivia* (9). Besides *nimK*, Tn*6456* also contains the genes for QacE family quaternary ammonium compound efflux small multidrug resistance transporter and Crp/Fnr family transcriptional regulator and genes for the insertion sequence (IS), mobilization protein, and integrase.

**Fig 2:**
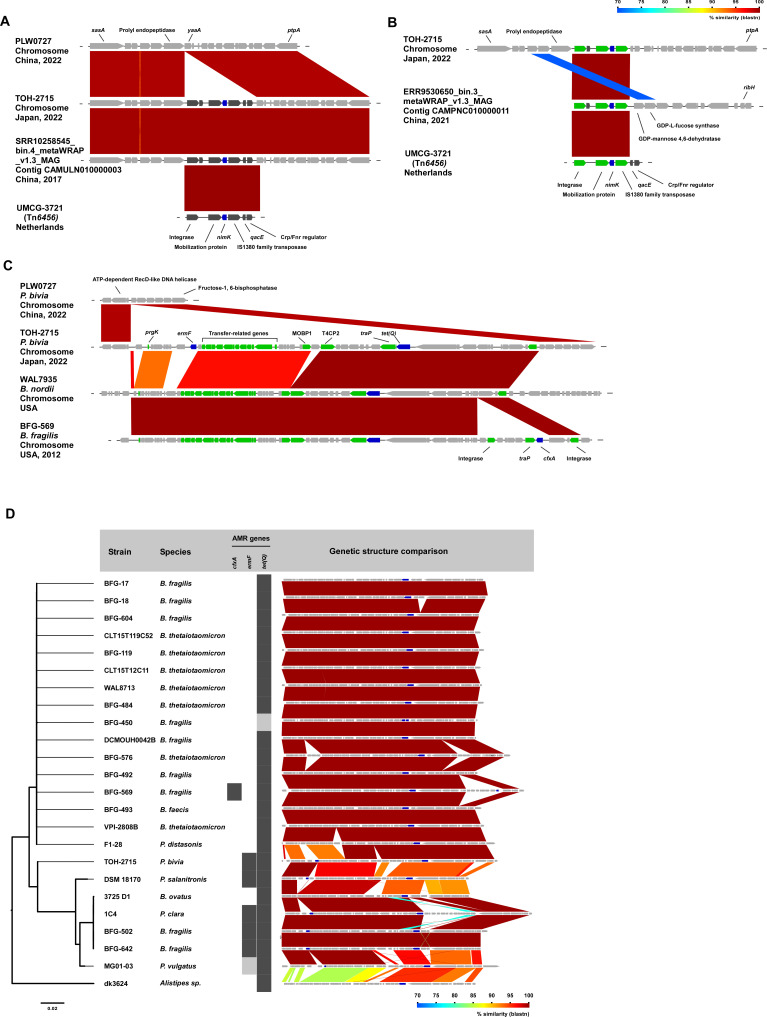
Comparative analysis of the MGE of TOH-2715. (**A**) MGE region 1 (Tn*6456*) of TOH-2715 was compared with PLW0727, SRR10258545 bin.4_metaWRAP_v1.3_MAG, and the insertion points of transposons and each gene were described. (**B**) Comparative analysis of the Tn6456-like element possessed by ERR9530650_bin.3_metaWRAP_v1.3_MAG and Tn*6456* was performed and depicted. (**C**) MGE region 2 (ICE) of TOH-2715 was compared with PLW0727, WAL7935, and BFG-569 strains, and the insertion sites of ICEs and each gene were described. Green panels indicate conjugation- and mobilization-related genes, and blue panels indicate AMR genes. (**D**) For MGE region 2, phylogenetic analysis was performed based on core ICE genes collected from similar ICE (CTnDOT-family) from public databases. Black panels indicate the presence of antimicrobial resistance genes, and gray panels indicate AMR genes that are broken.

Comparison of the genetic structure of TOH-2715 MGE-region 1 harboring *nimK* with Tn*6456* revealed a sequence match of 7,820 out of 7,821 bp. A Blastn search also identified another contig containing Tn*6456*, which was registered in China in 2017 (accession no. CAMULN010000003) in addition to *P. bivia* strain UMCG-3721. With the exception of UMCG-3721, for which WGS data could not be retrieved, chromosomal insertion sites for TOH-2715 and the contig of SRR10258545_bin.4_metaWRAP_v1.3_MAG were confirmed as similarly located between the prolyl endopeptidase gene and *yaaA* (Fig. 2A). We further detected *nimK* in the metagenome assembly contig of ERR9530650_bin.3_metaWRAP_v1.3_MAG, registered in China in 2021, which showed 94.8% identity with *Hallella colorans* (formerly *P. colorans*, DSM 100333^T^, accession no. QENY00000000.1) by ANI analysis and was considered a different species from *P. bivia*. Comparison of the structure surrounding *nimK* in this strain with that of Tn*6456* revealed the presence of a Tn*6456*-like element lacking the efflux transporter gene *qacE* and the Crp/Fnr regulator gene, demonstrating the existence of a variant of Tn*6456* (Fig. 2B). Although the upstream structure of the integrase gene is unknown because the genome assembly is at contig level, GDP-mannose 4,6-dehydratase is present downstream of the IS*1380* family transposase, which is thought to be a different insertion site than that in strain TOH-2715. Meanwhile, the nucleotide sequence of *nimK* was a complete match in all four strains.

While *nim* has a low prevalence, *tet*(Q) and *ermF* are relatively highly prevalent in important anaerobic bacteria belonging to *Bacteroides* and *Prevotella* (13). In *Bacteroides*, *tet*(Q) and *ermF* are located on the CTn*DOT* and CTn*DOT*-family transposons, but their location in *Prevotella* spp. remains unknown (14). Therefore, we analyzed the location of these AMR genes by comparison with MGEs in other species, such as *Bacteroides* spp. and *Prevotella* spp., based on WGS data.

*ermF* and *tet*(Q) were present in MGE-region 2 (Fig. 1A). On the basis of the results of Alien Hunter analysis, the 58.8 Kbp region containing these genes was inferred as an MGE region. The GC content of the integrative and conjugative element (ICE) in TOH-2715 was 48.2%, suggesting that it is an exogenous element that was acquired by horizontal gene transfer (HGT) (Fig. 2C). The structure of MGE-region 2 of TOH-2715 was found to be highly comparable with the CTn*DOT*-family transposon (Fig. S1). A Blastn search revealed that *Bacteroides nordii* and *Bacteroides fragilis* had even more similar regions (Fig. 2C). The MGE-region 2 of TOH-2715 was found to harbor integrase and relaxase genes as well as type IV secretion system component genes, strongly suggesting an ICE. Phylogenetic analysis of 24 ICEs with coverages >80% detected in a Blast search revealed that the TOH-2715 ICE and ICEs from other species formed one large cluster (Fig. 2D).

Although the phylogenetic tree shows that this large cluster forms two subclusters, sequence comparison of these ICEs revealed that the CDS in the ICEs was also highly conserved. Because these ICEs form a large cluster in the phylogenetic analysis and have a similar genetic structure, this suggests the occurrence of HGT in anaerobic bacteria, including *P. bivia*.

Some ICEs, including that of TOH-2715, contained *ermF* in addition to *tet*(Q) (Fig. 2D). *tet*(Q) encodes Tet(Q), a tetracycline resistance determinant that confers resistance through a ribosome protection mechanism. *ermF* encodes a protein responsible for CLI resistance. CLI is an important treatment option for anaerobic bacteria, and the presence of AMR genes may complicate and render the treatment of *Prevotella* infections in clinical settings. The ICE of *B. fragilis* strain BFG-569 contained *cfxA*, which encodes class A β-lactamase, originally identified in *Bacteroides vulgatus* (Fig. 2D).

Because WGS-based data on the resistome of *P. bivia* are limited, we next attempted analysis using WGS data of TOH-2715 and 25 *P*. *bivia* strains retrieved from the Bacterial and Viral Bioinformatics Resource Center database (Fig. 1B). The abundance of *cfxA* (6/26) and its variant cfxA3 β-lactamase (10/26) was 62% (16/26). Relatively high abundances of *erm*F and *tet*(Q) were also observed, which were consistent with previous PCR-based results on the prevalence of AMRs, but the abundance of *ermF* was higher than previously reported (13, 15). In contrast to the relative abundance of these genes in *P. bivia*, *nimK*, *mef*(A), and *msr*(D) were each only found in 2 of the 26 strains (Fig. 1B).

The introduction of MGEs, such as ISs from the host strain genome, was reported to modify ICEs, which were subsequently transferred to other members of the microflora (16). Our study implicates the HGT of ICEs between *P. bivia* and anaerobic bacteria, including *Bacteroides* spp. Although Tn*6456* itself does not possess any genes involved in conjugation, there is concern that TOH-2715 ICE may be modified via intragenomic transitions, and the potential for HGT of *nimK* to *Prevotella* spp. and *Bacteroides* spp. is worrying.

The frequency of MTZ-resistant anaerobic bacteria in Japan remains unclear due to the lack of AMR surveillance of anaerobic bacteria. This report on the emergence of a clinical isolate of *P. bivia* carrying *nimK* highlights the need for caution regarding MTZ resistance in anaerobic bacteria in the future.

## Data Availability

The complete genome sequence of the *P. bivia* TOH-2715 strain has been deposited in GenBank under accession number SAMD00746874.

## SUPPLEMENTAL MATERIAL

Figure S1 spectrum.00562-24-s0001.docx Genomic structure comparison of CTnDOT-family transposon and the MGE region of TOH-2715.

Supplemental material spectrum.00562-24-s0002.docx WGS analysis methodology and information.

ASM does not own the copyrights to Supplemental Material that may be linked to, or accessed through, an article. The authors have granted ASM a non-exclusive, world-wide license to publish the Supplemental Material files. Please contact the corresponding author directly for reuse.

## References

[1] Könönen E, Gursoy UK. 2021. Oral Prevotella species and their connection to events of clinical relevance in gastrointestinal and respiratory tracts. Front Microbiol 12:798763. doi:10.3389/fmicb.2021.798763 35069501 PMC8770924

[2] Löfmark S, Edlund C, Nord CE. 2010. Metronidazole is still the drug of choice for treatment of anaerobic infections. Clin Infect Dis 50 Suppl 1:S16–23. doi:10.1086/647939 20067388

[3] Katsandri A, Avlamis A, Pantazatou A, Houhoula DP, Papaparaskevas J. 2006. Dissemination of nim-class genes, encoding nitroimidazole resistance, among different species of Gram-negative anaerobic bacteria isolated in Athens, Greece. J Antimicrob Chemother 58:705–706. doi:10.1093/jac/dkl285 16867996

[4] Wybo I, Van den Bossche D, Soetens O, Vekens E, Vandoorslaer K, Claeys G, Glupczynski Y, Ieven M, Melin P, Nonhoff C, Rodriguez-Villalobos H, Verhaegen J, Piérard D. 2014. Fourth Belgian multicentre survey of antibiotic susceptibility of anaerobic bacteria. J Antimicrob Chemother 69:155–161. doi:10.1093/jac/dkt344 24008826 PMC3861333

[5] Breuil J, Dublanchet A, Truffaut N, Sebald M. 1989. Transferable 5-nitroimidazole resistance in the Bacteroides fragilis group. Plasmid 21:151–154. doi:10.1016/0147-619x(89)90060-7 2740453

[6] Haggoud A, Reysset G, Azeddoug H, Sebald M. 1994. Nucleotide sequence analysis of two 5-nitroimidazole resistance determinants from Bacteroides strains and of a new insertion sequence upstream of the two genes. Antimicrob Agents Chemother 38:1047–1051. doi:10.1128/AAC.38.5.1047 8067736 PMC188148

[7] Alauzet C, Lozniewski A, Marchandin H. 2019. Metronidazole resistance and nim genes in anaerobes: a review. Anaerobe 55:40–53. doi:10.1016/j.anaerobe.2018.10.004 30316817

[8] Sevillano G, Miño APY, Solís MB, Vaca JP, Zurita-Salinas C, Zurita J. 2023. First report of antibiotic resistance markers cfiA and nim among bacteroides fragilis group strains in ecuadorian patients. Microb Drug Resist 29:533–539. doi:10.1089/mdr.2023.0125 37733248

[9] Veloo ACM, Chlebowicz M, Winter HLJ, Bathoorn D, Rossen JWA. 2018. Three metronidazole-resistant Prevotella bivia strains harbour a mobile element, encoding a novel nim gene, nimK, and an efflux small MDR transporter. J Antimicrob Chemother 73:2687–2690. doi:10.1093/jac/dky236 29982676 PMC6148209

[10] European Committee on Antimicrobial Susceptibility Testing (EUCAST). 2020. Breakpoint tables for interpretation of MICs and zone diameters. Version10.0. Available from: http://www.eucast.org

[11] Trinh S, Reysset G. 1996. Detection by PCR of the nim genes encoding 5-nitroimidazole resistance in Bacteroides spp. J Clin Microbiol 34:2078–2084. doi:10.1128/jcm.34.9.2078-2084.1996 8862561 PMC229193

[12] Peng Y, Cai X, Li M, Deng L, Wang Y, Qiu Y, Zhao L, Xiao Y, Xu L, Hou Q. 2023. The first completed genome of species Prevotella bivia, assembled from a clinically derived strain PLW0727. J Glob Antimicrob Resist 35:268–270. doi:10.1016/j.jgar.2023.10.009 37866682

[13] Veloo ACM, Baas WH, Haan FJ, Coco J, Rossen JW. 2019. Prevalence of antimicrobial resistance genes in Bacteroides spp. and Prevotella spp. Dutch clinical isolates. Clin Microbiol Infect 25:1156. doi:10.1016/j.cmi.2019.02.017 30802650

[14] Boiten KE, Kuijper EJ, Schuele L, van Prehn J, Bode LGM, Maat I, van Asten SAV, Notermans DW, Rossen JWA, Veloo ACM. 2023. Characterization of mobile genetic elements in multidrug-resistant Bacteroides fragilis isolates from different hospitals in the Netherlands. Anaerobe 81:102722. doi:10.1016/j.anaerobe.2023.102722 37001724

[15] Yokoyama S, Hayashi M, Goto T, Muto Y, Tanaka K. 2023. Identification of cfxA gene variants and susceptibility patterns in β-lactamase-producing Prevotella strains. Anaerobe 79:102688. doi:10.1016/j.anaerobe.2022.102688 36580990

[16] Coyne MJ, Zitomersky NL, McGuire AM, Earl AM, Comstock LE. 2014. Evidence of extensive DNA transfer between Bacteroidales species within the human gut. mBio 5:e01305-14. doi:10.1128/mBio.01305-14 24939888 PMC4073490

