# Supplementary material for: The emergence of metronidazole-resistant Prevotella bivia harboring nimK gene in Japan
Source: Microbiol Spectr. 2024 Aug 20;12(10):e00562-24. doi: 10.1128/spectrum.00562-24 (PMC11448248; doi:10.1128/spectrum.00562-24)
Supplement: Figure S1 — Genomic structure comparison of CTnDOT-family transposon and the MGE region of TOH-2715. [file spectrum.00562-24-s0001.docx]

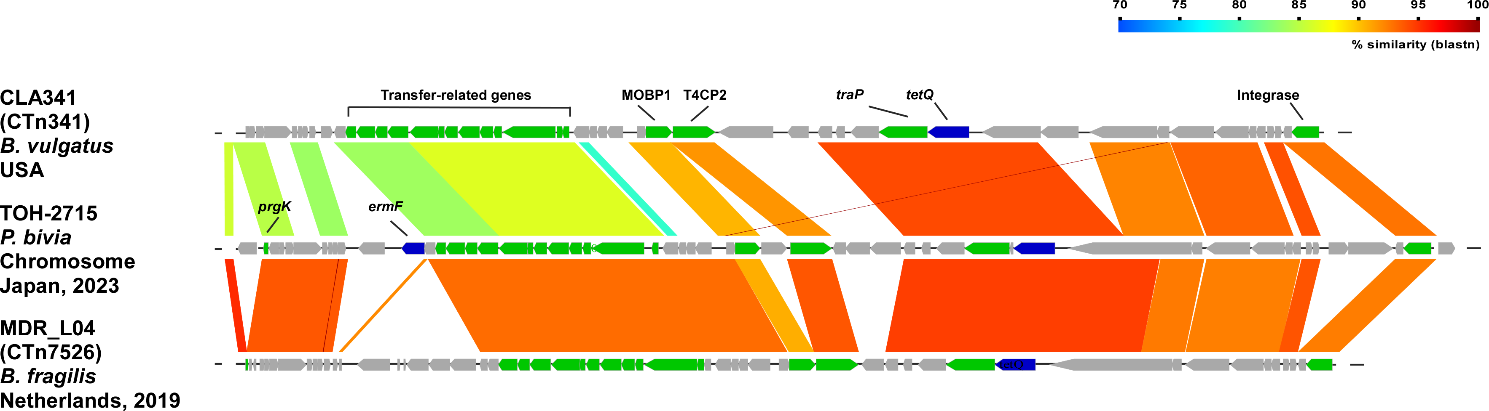


**Supplementary Figure 1**

**Genomic structure comparison of CTn*DOT*-family transposon and the MGE region of TOH-2715.**

The nucleotide sequence of MGE region 2 of TOH-2715 was compared with those of CTn*341* of *B. vulgatus* (accession number; AY515263) and CTn*7526* of *B. fragilis* (accession number; OP227219).
