# Supplementary material for: The emergence of metronidazole-resistant Prevotella bivia harboring nimK gene in Japan
Source: Microbiol Spectr. 2024 Aug 20;12(10):e00562-24. doi: 10.1128/spectrum.00562-24 (PMC11448248; doi:10.1128/spectrum.00562-24)
Supplement: Supplemental material — WGS analysis methodology and information. [file spectrum.00562-24-s0002.docx]

**Supplementary data**

**WGS analysis**

Genomic DNA was extracted using a Gentra Puregene Tissue Kit (Qiagen, Hilden, Germany) according to the manufacturer’s protocol. The whole genome sequencing (WGS) was performed on DNBSEQ (MGI, Shenzhen, China) and MinION (Oxford Nanopore Technologies, Oxford, UK). The DNA library for MinION sequencing was prepared using the Rapid Barcoding Kit according to the manufacturer's protocol and sequenced on an R9.4.1 flow cell (Oxford Nanopore Technologies, Oxford, UK). ONT long reads were base-called using Guppy v3.4.4, de novo assembled with Canu v2.1.1 (https://github.com/marbl/canu), and polished by MGI short paired-end reads using Pilon v1.20.1 (<https://github.com/broadinstitute/pilon>). Subsequently, the assembled sequence was submitted to the RAST (https://rast.nmpdr.org) and DFAST (https://dfast.ddbj.nig.ac.jp) pipelines to obtain gene annotations. Average nucleotide identity (ANI) with prokaryotic RefSeq genome sequences was obtained by the quality check option in the DFAST server.

Core genome analysis was performed using Roary v3.13.0 (https://github.com/sanger-pathogens/Roary), and minimum identity percentages of 70% for ICE and 95% for whole genome comparison were used for blastp. Phylogenetic trees were created using RAxML v8.2.12 (https://github.com/stamatak/standard-RAxML) with 1000 replicates of bootstrap, and visualized using Figtree v1.4.4 (<http://tree.bio.ed.ac.uk/software/figtree/>). Linear sequence comparison was performed using GenomeMatcher v3.01 (<http://www.ige.tohoku.ac.jp/joho/gmProject/gmhome.html>). The circular genome map was generated using Proksee (<https://proksee.ca>). Horizontal gene transfer-related regions were identified using Alien Hunter, and antimicrobial resistance genes were detected using the Comprehensive Antibiotic Resistance Database (CARD) library in Proksee. The Bacterial and Viral Bioinformatics Resource Center (BV-BRC) database (<https://www.bv-brc.org>) was used to obtain publicly available genome sequences of *Prevotella* spp..
